# Factors associated with gravity-dependent distribution on chest CT in elderly patients with community-acquired pneumonia: a retrospective observational study

**DOI:** 10.1038/s41598-022-12092-w

**Published:** 2022-05-16

**Authors:** Kosaku Komiya, Takashi Yamamoto, Hiroki Yoshikawa, Akihiko Goto, Kenji Umeki, Takeshi Johkoh, Kazufumi Hiramatsu, Jun-ichi Kadota

**Affiliations:** 1 Department of Respiratory Medicine and Infectious Diseases, Oita University Faculty of Medicine, 1-1 Idaigaoka, Hasama-machi, Yufu, Oita 879-5593 Japan; 2 Department of Internal Medicine, Tenshindo Hetsugi Hospital, 5956 Nihongi, Nakahetsugi, Oita 879-7761 Japan; 3 Kinki Central Hospital of Mutual Aid Association of Public School Teachers, 3-1 Kurumazuka, Itami, Hyogo 664-8533 Japan; 4 Medical Safety Management, Oita University Faculty of Medicine, 1-1 Idaigaoka, Hasama-machi, Yufu, Oita 879-5593 Japan

**Keywords:** Medical research, Risk factors

## Abstract

Although lung involvement in aspiration pneumonia typically has a gravity-dependent distribution on chest images, which patient’s conditions contribute to its radiological pattern has not been fully elucidated. This study was designed to determine the factors associated with the gravity-dependent distribution of community-acquired pneumonia (CAP) on chest computed tomography (CT). This retrospective study included elderly patients aged ≥ 65 years with CAP who underwent chest CT within 1 week before or after admission. The factors associated with lower lobe- and posterior-predominant distributions of ground glass opacity or airspace consolidation were determined. Of the 369 patients with CAP, 348 (94%) underwent chest CT. Multivariate analyses showed that impaired consciousness, a low Barthel index of activities of daily living, and high hemoglobin levels were associated with lower lobe-predominant distribution, while male sex and impaired consciousness were associated with posterior-predominant distribution. Cerebrovascular diseases were unrelated to these distributions. While male sex, impaired consciousness, high hemoglobin levels, low albumin levels, and the number of involved lobes were associated with in-hospital mortality, gravity-dependent distributions were not. Impaired consciousness might be the most significant predictor of aspiration pneumonia; however, the gravity-dependent distribution of this disease is unlikely to affect disease prognosis.

## Introduction

The incidence and mortality from pneumonia are increasing as the world population ages. A better understanding of how pneumonia develops is crucial to control morbidity and mortality in elderly patients. Aspiration is a major contributing factor for pneumonia development in the elderly^1,2^, and aspiration pneumonia occurs in individuals at risk of aspiration due to a swallowing disability caused by advanced age, impaired consciousness, or cerebrovascular disease^1^. However, how these or other conditions contribute to the development of aspiration pneumonia remains unknown.

Furthermore, there are no gold standard diagnostic criteria for aspiration pneumonia. Most clinical studies have defined aspiration pneumonia as pneumonia that occurs in patients with aspiration risks and/or gravity-dependent distribution on chest images^3^. Since aspirated materials fall with the force of gravity, it is reasonable that radiological images in aspiration pneumonia may show a gravity-dependent distribution^1,4,5^. This finding suggests aspiration-related pneumonia. However, detecting abnormal infiltrations using chest X-rays is challenging in elderly patients with pneumonia. In fact, chest X-ray-negative pneumonia has been reported in approximately 30% of healthcare associated pneumonia cases confirmed by chest computed tomography (CT)^6^.

Japan has the longest life expectancy in the world. Since universal health insurance fully covers chest CT when physicians suspect pneumonia, almost all elderly patients undergo chest CT on admission. Using this advantage, we conducted this study to determine the risk factors for aspiration pneumonia by assessing factors associated with gravity-dependent distribution on chest CT in elderly patients with community-acquired pneumonia (CAP). Gravity-dependent distribution may not perfectly denote aspiration pneumonia; however, no gold standard criteria for diagnosing aspiration pneumonia exist. Objectively evaluating which patient’s conditions contribute to the development of aspiration pneumonia is required for better management of elderly patients who are prone to pneumonia. Therefore, this study was designed to assess the factors associated with gravity-dependent distribution on chest CT in elderly patients with CAP.

## Patients and methods

### Patients and study design

This retrospective observational study was conducted at our community hospital with 188 beds in Oita Prefecture, Japan. CAP was defined based on the criteria of the American Thoracic Society/Infectious Diseases Society of America^7^. In brief, the disease was diagnosed using clinical signs and symptoms, including cough and fever, and infiltrates revealed on chest radiography or chest CT. We included consecutive patients (age ≥ 65) admitted to the hospital between January 2017 and May 2018 for CAP and those who had undergone chest CT within 1 week before or after admission. Patients with pulmonary tuberculosis; non-bacterial pneumonia, including interstitial pneumonia or fungal infections; and cardiac pulmonary edema and those treated with immunosuppressants were excluded.

This study was designed to determine factors associated with gravity-dependent distribution on chest CT. Gravity-dependent distribution was evaluated as “lower lobe-predominance” and “posterior-predominance” on chest CT. As a secondary endpoint, the impact of these distributions on in-hospital mortality was analyzed.

The study protocol was approved by the Institutional Ethics Committee of Tenshindo Hetsugi Hospital (Approval Number: 2171, Approval Date: December 20, 2021). The committee waived informed consent because of the retrospective nature of the study. Some patients were included in previous studies^8,9^.

### Data collection

Patient admission data included sex, age, body mass index (BMI), oxygenation status, consciousness level, activities of daily living (ADL), comorbidities, and laboratory data (i.e., white blood cell count, hemoglobin, C-reactive protein levels, albumin levels, and aspartate aminotransferase activity). These tests are routinely documented when a patient diagnosed with CAP is admitted. Impaired consciousness was defined as scores of ≤ 13 in the Glasgow Coma Scale, and respiratory failure was defined as a SpO_2_ of < 90% without supplemental oxygen on admission. The ADL level before admission was assessed with the Barthel index. The Barthel index was introduced in 1965 and originally used a 0–20 scale^10^. It was modified by Granger et al. in 1979^11^ to include 0–10 points for each item with a total possible score of 0–100. Information relating to the 10 basic ADL are collected with the revised Barthel index: bowel, bladder, grooming, toilet use, feeding, transfers, walking, dressing, climbing stairs, and bathing. Sputum culture results were also obtained from medical records.

### Evaluation of chest CT findings

A 320-detector row CT system (AquilionONE; Toshiba Medical Systems, Tokyo, Japan) was used. Scans were obtained with contiguous 2.0-mm-thick sections from the apex to the lung base. Images were captured at a − 600 HU (level) and 1,500 HU (width) window setting.

Two respiratory medicine specialists with 12 and 18 years of experience, blinded to the patients’ clinical information, retrospectively assessed the presence of cavitary lesions and patchy shadows, and distribution of grand glass opacity (GGO) and airspace consolidation on chest CT. In addition to lobar distributions, cross-sectional locations were classified as “anterior” (in the ventral half of each lobe) or “posterior” (in the dorsal half of each lobe). As for the assessment of gravity-dependent distribution, we determined “lower lobe-predominant distribution” and “posterior-predominant distribution.” A review conducted by the same two physicians to reach a consensus resolved any disagreement between the presence and predominance of these findings in each case.

### Statistical analyses

Statistical analyses were performed using SPSS software version 22 (IBM Corp., Japan, Tokyo, Japan). For two-tailed analyses, 95% confidence intervals were calculated. Interobserver agreement was assessed by kappa value analysis. Included cases were divided into presence or absence of lower lobe-predominant distribution, and posterior-predominant distribution, respectively. Patient data, clinical characteristics, and laboratory data from patients in each group were compared according to each distribution pattern. We considered variables with a *P*-value < 0.05 in the univariate analysis as eligible for entry in the multivariate logistic regression analysis.

The Cox hazard regression model was applied to determine the association between distribution pattern and in-hospital mortality. Variables with a *P*-value of < 0.05 between non-survivors and survivors in the univariate analysis were entered in the multivariate analysis.

### Ethical approval

The study protocol was approved by the Institutional Ethics Committee of Tenshindo Hetsugi Hospital (Approval Number: 2171 Approval Date: 20 December 2021).

## Results

### Baseline characteristics

Of the 369 elderly patients admitted to our institution during the study period, 348 (94%) underwent chest CT 1 week before or after admission and were included in this study. Bacterial pathogens were isolated from 146 of the 348 patients (42%). The isolated pathogens included *Klebsiella pneumoniae* (n = 21), methicillin‐resistant *Staphylococcus aureus* (n = 34), *Streptococcus pneumoniae* (n = 18), *Pseudomonas aeruginosa* (n = 12), *Haemophilus influenzae* (n = 14), methicillin-susceptible *Staphylococcus aureus* (n = 18), *Moraxella catarrhalis* (n = 10), *Escherichia coli* (n = 8), *Haemophilus parainfluenzae* (n = 2), *Klebsiella oxytoca* (n = 5), and *Prevotella* sp. (n = 5).

The median age was over 80 years, and almost half of the patients had respiratory failure. The median of the Barthel index before admission was 35; therefore, patients were divided into those with a high ADL (> 35) and a low ADL (≤ 35). The kappa values of the CT findings were 0.674 for cavitary lesion, 0.830 for patchy shadow, 0.827 for lower lobe-predominance, and 0.756 for posterior-predominance.

### Factors associated with lower lobe-predominant distribution

Patients with a lower lobe-predominant distribution had a higher proportion of impaired consciousness, a lower ADL level, and higher levels of neutrophil count and hemoglobin than those without lower lobe-predominant distribution (Table 1). No significant difference in risk factors that are considered for aspiration pneumonia, such as advanced age or cerebrovascular disease, was observed between patients with and without lower lobe-predominant distribution. Moreover, no difference in the frequency of cavitary lesions and patchy shadows was observed between these groups. In the multivariate analysis, impaired consciousness, a low ADL level, and a high hemoglobin level were significantly associated with lower lobe-predominant distribution after adjusting for neutrophil count (Table 2).

**Table 1:** Univariate logistic regression analysis of the baseline characteristics associated with lower lobe-predominant distribution.

	Lower lobe-predominance (n = 180)	Non- lower lobe-predominance (n = 168)	OR (95% CI)	*p*-value
Female	82 (46)	90 (54)	0.725 (0.476–1.106)	0.135
Age, yr	86 (80–90)	87 (81–92)	0.992 (0.967–1.017 )	0.527
BMI, kg/m^2^	18.4 (16.5–21.5)	19.0 (16.4–21.9)	0.981 (0.930–1.035)	0.487
Impaired consciousness	46 (26)	20 (12)	2.540 (1.430–4.513)	0.001
Systolic blood pressure, mmHg	119 (104–137)	120 (104–136)	0.998 (0.989–1.007)	0.643
Respiratory failure	66 (37)	86 (51)	0.973 (0.899–1.052)	0.488
Current or former smoker	39 (36)	47 (28)	1.490 (0.947–2.345)	0.084
Barthel index > 35	75 (42)	92 (55)	0.590 (0.386–0.902)	0.015
Chronic obstructive pulmonary disease	24 (13)	21 (13)	1.077 (0.575–2.017)	0.817
Heart failure	36 (20)	37 (22)	0.885 (0.528–1.483)	0.643
Cerebrovascular diseases	55 (31)	44 (26)	1.240 (0.777–1.980)	0.367
Diabetes mellitus	28 (16)	17 (10)	1.636 (0.860–3.113)	0.134
Malignancy	9 (5)	9 (5)	0.930 (0.360–2.401)	0.881
White blood cells, 10^3^/μL	9.3 (6.6–12.8)	9.2 (6.2–12.4)	1.000 (1.000–1.000)	0.666
Neutrophils, 10^3^/μL	8.5 (5.2–12.5)	7.4 (4.7–11.1)	1.000 (1.000–1.000)	0.049
Hemoglobin, g/dL	12.1 (10.8–13.4)	11.7 (10.3–12.7)	1.152 (1.032–1.286)	0.011
Aspartate aminotransferase, IU/L	22 (18–32)	23 (18–31)	1.005 (0.995–1.014)	0.343
eGFR, mL/min/1.73 m^2^	57.1 (39.5–86.9)	58.7 (37.1–79.6)	1.000 (0.995–1.005)	0.889
C–reactive protein, mg/dL	8.4 (3.2–15.5)	6.7 (2.8–13.6)	1.013 (0.987–1.041)	0.323
Albumin, g/dL	3.1 (2.8–3.5)	3.2 (2.8–3.6)	0.888 (0.619–1.273)	0.518
Cavitary lesion	11 (6)	12 (7)	0.846 (0.363–1.973)	0.699
Patchy shadow	57 (32)	69 (41)	0.665 (0.428–1.032)	0.069

Data are presented as number (%) or median (interquartile range).

BMI, body mass index; CI, confidence interval; eGFR, estimated glomerular filtration rate; OR, odds ratio.

**Table 2:** Multivariate logistic regression analysis of the baseline characteristics associated with lower lobe-predominant distribution.

	OR (95% CI)	*p*-value
Impaired consciousness	2.139 (1.154–3.966)	0.016
The Barthel index > 35	0.601 (0.375–0.965)	0.035
Neutrophils, 10^3^/μL	1.000 (1.000–1.000)	0.068
Hemoglobin, g/dL	1.183 (1.054–1.329)	0.004

CI, confidence interval; OR, odds ratio.

### Factors associated with posterior-predominant distribution

Patients with a posterior-predominant distribution had higher proportions of male sex and impaired consciousness and lower levels of BMI, ADL, and serum albumin (Table 3). Moreover, no significant difference in the proportion of patients with advanced age or cerebrovascular diseases was found between patients with and without posterior-predominant distribution. Furthermore, no difference in the frequency of cavitary lesions and patchy shadows was observed between the two groups. Multivariate analysis showed that male sex and impaired consciousness were significantly associated with posterior-predominant distribution after adjusting for BMI, ADL level, and albumin level (Table 4).

**Table 3:** Univariate logistic regression analysis of the baseline characteristics associated with posterior-predominant distribution.

	Posterior-predominance (n = 151)	Non-posterior-predominance (n = 197)	OR (95% CI)	*p*-value
Female	63 (42)	109 (55)	0.538 (0.377–0.887)	0.012
Age, yr	87 (81–90)	86 (80–92)	1.001 (0.976–1.027)	0.921
BMI, kg/m^2^	18.1 (16.2–20.9)	19.5 (16.8–22.6)	0.923 (0.872–0.977)	0.006
Impaired consciousness	43 (29)	23 (12)	3.012 (1.720–5.275)	< 0.001
Systolic blood pressure, mmHg	117 (103–133)	122 (106–139)	0.993 (0.984–1.002)	0.113
Respiratory failure	91 (60)	101 (51)	0.977 (0.907–1.053)	0.550
Current or former smoker	54 (36)	59 (30)	1.302 (0.829–2.045)	0.252
Barthel index > 35	57 (38)	110 (56)	0.480 (0.311–0.739)	< 0.001
Chronic obstructive pulmonary disease	19 (13)	26 (13)	0.947 (0.502–1.784)	0.865
Heart failure	28 (19)	45 (23)	0.769 (0.453–1.304)	0.330
Cerebrovascular diseases	50 (33)	49 (25)	1.495 (0.936–2.388)	0.092
Diabetes mellitus	25 (17)	20 (10)	1.756 (0.934–3.300)	0.080
Malignancy	9 (6)	9 (5)	1.324 (0.512–3.421)	0.562
White blood cells, 10^3^/μL	8.8 (6.1–12.4)	9.6 (6.5–12.7)	1.000 (1.000–1.000)	0.370
Neutrophils, 10^3^/μL	7.4 (5.0–12.2)	7.9 (5.1–11.6)	1.000 (1.000–1.000)	0.066
Hemoglobin, g/dL	11.6 (10.6–13.2)	12.0 (10.3–13.1)	1.032 (0.926–1.150)	0.566
Aspartate aminotransferase, IU/L	22 (18–31)	23 (18–32)	1.003 (0.994–1.013)	0.466
eGFR, mL/min/1.73 m^2^	56.8 (37.5–88.0)	58.9 (40.8–79.1)	1.003 (0.998–1.008)	0.218
C-reactive protein, mg/dL	8.9 (3.7–15.6)	6.6 (2.4–13.6)	1.017 (0.991–1.045)	0.200
Albumin, g/dL	3.1 (2.7–3.4)	3.3 (2.9–3.7)	0.581 (0.400–0.845)	0.005
Cavitary lesion	11 (7)	12 (6)	1.1211 (0.519–2.826)	0.657
Patchy shadow	60 (40)	66 (34)	1.309 (0.843–2.032)	0.231

Data are presented as number (%) or median (interquartile range).

BMI, body mass index; CI, confidence interval; eGFR, estimated glomerular filtration rate; OR, odds ratio.

**Table 4:** Multivariate logistic regression analysis of the baseline characteristics associated with posterior-predominant distribution.

	OR (95% CI)	*p*-value
Female	0.474 (0.296–0.758)	0.002
BMI, kg/m^2^	0.953 (0.896–1.014)	0.126
Impaired consciousness	2.385 (1.277–4.456)	0.006
Barthel index > 35	0.619 (0.373–1.029)	0.064
Albumin, g/dL	0.742 (0.492–1.118)	0.153

CI, confidence interval; eGFR, estimated glomerular filtration rate; OR, odds ratio.

### Distribution patterns and in-hospital mortality

The distribution patterns were not significantly associated with in-hospital mortality (Table 5). Instead, non-survivors had higher proportions of male sex and impaired consciousness, lower levels of hemoglobin and albumin, higher level of aspartate aminotransferase, and more involved lobes than survivors. In the multivariate analysis, male sex, impaired consciousness, low levels of hemoglobin and albumin, and several involved lobes were independently associated with in-hospital mortality (Table 6).

**Table 5:** Univariate Cox hazard regression analysis of the baseline characteristics and radiological features associated with in-hospital mortality.

	Non-survivor (n = 59)	Survivor (n = 289)	HR (95% CI)	*p*-value
Female	22 (37)	150 (52)	0.552 (0.323–0.944)	0.030
Age, yr	88 (84–92)	86 (79–91)	1.036 (1.000–1.073)	0.051
BMI, kg/m^2^	17.8 (15.0–20.3)	18.9 (16.9–21.9)	0.943 (0.878–1.013)	0.109
Impaired consciousness	21 (36)	45 (16)	2.110 (1.233–3.610)	0.006
Systolic blood pressure, mmHg	115 (100–131)	121 (104–139)	0.995 (0.985–1.006)	0.383
Respiratory failure	47 (80)	145 (50)	1.003 (0.938–1.072)	0.931
Current or former smoker	23 (39)	90 (31)	1.352 (0.797–2.292)	0.263
Barthel index > 35	20 (34)	147 (51)	0.666 (0.388–1.144)	0.141
Chronic obstructive pulmonary disease	6 (10)	39 (14)	0.847 (0.363–1.977)	0.702
Heart failure	18 (31)	55 (19)	1.213 (0.692–2.128)	0.500
Cerebrovascular diseases	20 (34)	79 (27)	1.057 (0.613–1.821)	0.843
Diabetes Mellitus	5 (9)	40 (14)	0.564 (0.225–1.413)	0.221
Malignancy	6 (10)	12 (4)	2.198 (0.941–5.133)	0.069
White blood cells, 10^3^/μL	8.7 (5.8–14.1)	9.2 (6.5–12.4)	1.000 (1.000–1.000)	0.536
Neutrophils, 10^3^/μL	7.2 (4.7–13.1)	7.9 (5.1–11.6)	1.000 (1.000–1.000)	0.688
Hemoglobin, g/dL	10.9 (9.8–12.1)	12.0 (10.7–13.2)	0.849 (0.749–0.963)	0.011
Aspartate aminotransferase, IU/L	26 (18–37)	22 (18–31)	1.010 (1.003–1.017)	0.007
eGFR, mL/min/1.73 m^2^	54.3 (32.7–93.7)	58.3 (38.5–80.1)	1.001 (0.995–1.006)	0.810
C-reactive protein, mg/dL	7.7 (3.9–17.2)	7.6 (2.9–14.0)	1.010 (0.981–1.039)	0.514
Albumin, g/dL	2.8 (2.2–3.2)	3.3 (2.9–3.6)	0.325 (0.205–0.515)	< 0.001
Lower lobe-predominance	30 (51)	150 (52)	0.745 (0.443–1.252)	0.266
Posterior-predominance	30 (51)	121 (42)	1.098 (0.654–1.841)	0.724
Cavitary lesion	7 (12)	16 (6)	1.415 (0.631–3.173)	0.399
Patchy shadow	25 (42)	101 (35)	1.272 (0.754–2.147)	0.367
Number of involved lobes	4 (3–5)	3 (2–5)	1.207 (1.008–1.446)	0.041

Data are presented as the number (%) or median (interquartile range).

BMI, body mass index; CI, confidence interval; eGFR, estimated glomerular filtration rate; HR, hazard ratio.

**Table 6:** Multivariate Cox hazard regression analysis of the baseline characteristics associated with in-hospital mortality.

	HR (95% CI)	*p*-value
Female	0.449 (0.237–0.814)	0.009
Impaired consciousness	2.353 (1.279–4.327)	0.006
Hemoglobin, g/dL	0.851 (0.733–0.989)	0.035
Aspartate aminotransferase, IU/L	1.002 (0.994–1.011)	0.610
Albumin, g/dL	0.471 (0.286–0.777)	0.003
Number of involved lobes	1.229 (1.019–1.482)	0.031

CI, confidence interval; HR: hazard ratio.

## Discussion

In this study, impaired consciousness was independently associated with both lower lobe-predominant and posterior-predominant distributions among elderly patients with CAP. However, these gravity-dependent distributions were unrelated to in-hospital mortality.

The result that impaired consciousness was associated with gravity-dependent distribution reflects that consciousness level may be the most significant predictor of aspiration pneumonia. The proportion of patients with cerebrovascular diseases, which are considered as a risk factor for aspiration^1^, were not significantly different between patients with and without these gravity-dependent distribution. Impaired consciousness might confound between cerebrovascular diseases and the risk of aspiration pneumonia. Not only the presence or absence of cerebrovascular diseases but also its severity affects the risk of aspiration pneumonia^12,13^. It is reasonable to assume that severe cerebrovascular diseases eventually deteriorate a patient’s condition, impairing levels of consciousness and ADL^14^, which may increase the risk of aspiration. A low ADL level was independently associated with lower lobe-predominance but not with posterior-predominance. While the ADL level might be related to the development of aspiration pneumonia, its impact seems less substantial than impaired consciousness.

Along with impaired consciousness and low ADL levels, a high hemoglobin level was associated with lower lobe-predominant distribution in the multivariate analysis. A high hemoglobin level generally indicates a good trophic condition. Patients maintaining better nutritional status are unlikely to have a risk for aspiration because they are anticipated to intake sufficient nutrition^15^. Indeed, another scale for nutrition, the BMI, did not differ between patients with and without lower lobe-predominant distribution. One possible reason might be the effect of dehydration. Fluid deprivation can lead to a relative increase in hemoglobin, and could be a risk for aspiration pneumonia because of poor oral hygiene and pathogen aspiration^16^.

Male sex was a significantly associated predictive factor for posterior-predominant distribution. It is unclear how male sex can contribute to the distribution. Still, women are more likely to be affected by middle-lung lobe syndrome^17^, which distributes to the anterior lung field on cross-sectional evaluation. This finding might bias the determination of posterior-predominance.

The main finding of this study was that impaired consciousness was independently associated with gravity-dependent distribution among elderly patients with CAP. In contrast, these gravity-dependent distributions were not associated with in-hospital mortality. Instead, male sex, impaired consciousness, low levels of hemoglobin and albumin, and several involved lobes were independently related to mortality. Assuredly, gravity-dependent distribution may not be a poor prognostic factor, and the results of the multivariate analysis of the baseline characteristics associated with in-hospital mortality implied that gravity-dependent distribution is confounded by male sex, impaired consciousness, higher levels of hemoglobin and albumin, and extensive pneumonia. In fact, these factors have been documented as poor prognostic factors for CAP^18–20^.

We previously published a systematic review showing that the diagnosis of aspiration pneumonia was associated with poor prognosis among patients with CAP^21^. The results in the current study seem to be contradictory. However, the current study focused on the gravity-dependent distribution pattern, and it may not absolutely be equal to the true diagnosis of aspiration pneumonia. Some patients with non-aspiration pneumonia may show gravity-dependent distribution on radiological images. Conversely, aspiration pneumonia cannot be denied based on the distribution pattern of pneumonia. For example, severe aspiration pneumonia could show diffuse GGO or airspace consolidation, which does not always take gravity-dependence. Indeed, patients with more lobe involvement were independently associated with in-hospital mortality in this study. Regardless of whether pneumonia is caused by aspiration, severe pneumonia is less likely to show gravity-dependent distribution on chest images^22^.

Nevertheless, the evaluation of these gravity-dependent distributions remains worth considering to suspect aspiration pneumonia as a mechanism of pneumonia development. The purpose of diagnosing aspiration pneumonia is not only to predict prognosis but also to suggest prevention strategies for aspiration of oral secretions through aggressive oral care and food texture modification, among others. These changes might improve disease progression and avoid recurrence^23–25^.

The strength of this study is that almost all patients underwent chest CT on admission, and ADL level was routinely assessed before admission using the Barthel index in addition to the collection of general patients’ information, including consciousness level, in our hospital. However, study limitations need to be discussed. First, this study was conducted at a single hospital, and the median of included patients’ age was over 80. The results in this study might not be generalizable in elderly patients over 65, but in many countries, life expectancy is estimated to be longer^26^. Second, evaluation of the distribution predominance on chest CT by each respiratory medicine specialist was not perfectly consistent. Nevertheless, kappa values were moderate to good. Third, GGO or airspace consolidation observed on chest CT might not denote an acute inflammatory reaction in older adults^27^. The evaluation of a past chest CT or a follow-up chest CT after treatment is required to solve this issue, but we could not confirm due to its retrospective nature. Finally, as discussed, gravity-dependent distribution does not absolutely denote “aspiration pneumonia.” However, no objective diagnostic criteria for aspiration pneumonia currently exist. Gravity-dependent distribution is one of the suggestive features of aspiration pneumonia, and this study revealed the factors that increase the risk of aspiration. No studies focusing on this association have been published.

In conclusion, impaired consciousness rather than cerebrovascular diseases or a low ADL level was significantly associated with gravity-dependent lung involvement on chest CT in elderly patients with CAP. The consciousness level might be a major predictive factor for aspiration pneumonia. To validate these results, a prospective study to determine whether a preventive intervention against oral secretion aspiration is effective for pneumonia recurrence among elderly patients with CAP showing gravity-dependent distribution is necessary.
